# Dietary Crude Lecithin Increases Systemic Availability of Dietary Docosahexaenoic Acid with Combined Intake in Rats

**DOI:** 10.1007/s11745-016-4139-8

**Published:** 2016-04-01

**Authors:** Nick van Wijk, Martin Balvers, Mehmet Cansev, Timothy J. Maher, John W. C. Sijben, Laus M. Broersen

**Affiliations:** Nutricia Research, Nutricia Advanced Medical Nutrition, PO Box 80141, 3508 TC Utrecht, The Netherlands; Department of Pharmacology, Uludag University Medical School, Gorukle, 16059 Bursa, Turkey; Department of Pharmaceutical Sciences, MCPHS University, 179 Longwood Avenue, Boston, MA 02115 USA; Department of Brain and Cognitive Sciences, Massachusetts Institute of Technology, Cambridge, MA USA; Utrecht Institute for Pharmaceutical Sciences (UIPS), Utrecht University, Utrecht, The Netherlands

**Keywords:** Phospholipids, Docosahexaenoic acid, Plasma lipids, n-3 Fatty acids, Nutrition, Fish oil, Algal lipids

## Abstract

Crude lecithin, a mixture of mainly phospholipids, potentially helps to increase the systemic availability of dietary omega-3 polyunsaturated fatty acids (n-3 PUFA), such as docosahexaenoic acid (DHA). Nevertheless, no clear data exist on the effects of prolonged combined dietary supplementation of DHA and lecithin on RBC and plasma PUFA levels. In the current experiments, levels of DHA and choline, two dietary ingredients that enhance neuronal membrane formation and function, were determined in plasma and red blood cells (RBC) from rats after dietary supplementation of DHA-containing oils with and without concomitant dietary supplementation of crude lecithin for 2–3 weeks. The aim was to provide experimental evidence for the hypothesized additive effects of dietary lecithin (not containing any DHA) on top of dietary DHA on PUFA levels in plasma and RBC. Dietary supplementation of DHA-containing oils, either as vegetable algae oil or as fish oil, increased DHA, eicosapentaenoic acid (EPA), and total n-3 PUFA, and decreased total omega-6 PUFA levels in plasma and RBC, while dietary lecithin supplementation alone did not affect these levels. However, combined dietary supplementation of DHA and lecithin increased the changes induced by DHA supplementation alone. Animals receiving a lecithin-containing diet also had a higher plasma free choline concentration as compared to controls. In conclusion, dietary DHA-containing oils and crude lecithin have synergistic effects on increasing plasma and RBC n-3 PUFA levels, including DHA and EPA. By increasing the systemic availability of dietary DHA, dietary lecithin may increase the efficacy of DHA supplementation when their intake is combined.

## Introduction

Omega-3 polyunsaturated fatty acids (n-3 PUFA), such as docosahexaenoic acid (DHA), are important structural components of neuronal membranes and are functionally implied in various membrane-bound processes [1, 2]. Dietary supplementation of n-3 PUFA raises blood n-3 PUFA levels rapidly [3, 4] and increases their uptake into the brain and therefore their availability for incorporation into neuronal membranes [5, 6]. Thus, neuronal membrane structure and function are subject to alterations induced by nutritional compounds such as n-3 PUFA.

As a result of its biochemical and biophysical properties, dietary lecithin may help to increase the availability of dietary n-3 PUFA, with subsequent effects on neuronal membrane structure and function. Crude lecithin is a mixture of mainly phospholipids (up to approximately 75 % for de-oiled lecithin), i.e., phosphatidylcholine (PtdCho), phosphatidylethanolamine (PtdEtn), and phosphatidylinositol (PtdIns), and a smaller fraction of glycolipids, neutral lipids, and carbohydrates, that can be extracted from plant or animal food substances. Lecithin has low solubility in water, but is a very good emulsifier and is also a source of active compounds, such as choline, that can be released from PtdCho. Theoretically, dietary lecithin (i.e., phospholipids) could increase systemic availability of dietary n-3 PUFA, since (biliary) phospholipids are required for the absorption of fat from the gut lumen into the enterocytes and the lymph [7, 8]. Several preclinical studies have examined the potential additive effects of lecithin or phospholipids on the bioavailability of triglycerides after oral intake [9–15]. However, most of these studies investigated (1) post-prandial effects of a single dose and/or (2) effects on lymphatic accretion and/or (3) effects of emulsification, i.e., whether pre-emulsifying oil with lecithin enhances the oil’s bioavailability. For example, recently Couëdelo *et al*. showed in rats that the lymphatic accretion after single intra-gastric administration of an oil containing the omega-3 fatty acid alpha-linolenic acid (ALA) could be increased by pre-emulsifying the oil with lecithin [14]. No distinct data exist on the effects of prolonged combined dietary supplementation of n-3 PUFA, especially DHA, and phospholipids on plasma and red blood cell (RBC) PUFA levels. Systemic availability is functionally relevant as the availability of n-3 PUFA to tissues such as the brain is largely dependent on their levels in circulation.

The objective of the present study was to test the hypothesized additive effects of dietary crude lecithin (which itself does not contain DHA) on top of dietary supplementation with DHA-containing oils. Experimental diets were presented to rats for 2–3 weeks after which relative and quantitative plasma levels and relative RBC levels of DHA, eicosapentaenoic acid (EPA), total n-3 PUFA, and total omega-6 (n-6) PUFA were determined. Plasma choline was measured to confirm that dietary lecithin, being a source of PtdCho, increases plasma choline concentration.

## Materials and Methods

To investigate the effects of dietary lecithin on the systemic availability of dietary DHA in adult rats, two experiments were conducted at the Centrum Kleine Proefdieren, Wageningen University (Wageningen, The Netherlands).

### Animals

Twenty-four male Wistar rats (HsdCpb:WU) (Experiment A) and 24 Sprague–Dawley rats (Hsd:SD) (Experiment B) were obtained from Harlan (Harlan Nederland, Horst, The Netherlands). Animals aged 10 weeks on arrival were housed in groups in a temperature- and light-controlled room, under 12 h light–12 h dark cycles. Rats had free access to experimental diets and water. Body weight and food-intake was recorded at least once a week. All experimental protocols were conducted in accordance with international and national laws and institutional guidelines and approved by the local ethics committee [DEC Consult, Bilthoven, The Netherlands, protocol numbers DEC Nr. NR117 (Experiment A) and DEC Nr. DAN0190 (Experiment B)].

### Diets

In Experiment A, four grain-based experimental diets with varying levels of vegetable algae DHA oil and crude lecithin were used: (1) control; (2) lecithin; (3) vegetable DHA oil; (4) vegetable DHA oil + lecithin. In Experiment B, three AIN-93M-based diets were used with varying levels of fish oil and crude lecithin: (1) control; (2) fish oil; (3) fish oil + lecithin. Within each experiment the diets were isocaloric and identical with respect to their protein, carbohydrate, fiber, and mineral contents. The diets fulfilled minimal dietary requirements for rats and were presented to the animals as pellets. The diets differed in composition with regard to the fat blends used, as well as the amount of lecithin added. The control diets and the lecithin diet did not contain DHA or EPA and the control diets did not contain any added crude lecithin. The lecithin-containing diets in Experiment A were supplemented with 400 mg de-oiled crude soy lecithin/100 g diet (0.4 %, Emulpur IP, Cargill Texturizing Solutions, The Netherlands), whereas the amount of de-oiled crude lecithin added to the lecithin-containing diet in Experiment B was 1000 mg/100 g diet (1.0 %). Average daily intake of crude lecithin per body weight was calculated based on the respective measured average body weight and food intake and was approximately 250 mg crude lecithin/kg BW/day (Experiment A) and 550 mg crude lecithin/kg BW/day (Experiment B). The de-oiled soy crude lecithin mainly contained phospholipids (77 g phospholipids/100 g lecithin) of which mainly PtdCho (20 g/100 g lecithin), PtdIns (14 g/100 g lecithin), and PtdEtn (13 g/100 g lecithin, as provided by the supplier), with mainly linoleic acid (18:2n-6), palmitic acid (16:0), and oleic acid (18:1n-9), and did not contain any DHA. The total fat content of the fish oil + lecithin diet in Experiment B was corrected for the fat content of the added lecithin. In Experiment A, the amount of fat from lecithin was considered to be negligible. The DHA content of the diets containing vegetable algae DHA oil (DHASCO oil, Martek Biosciences Corporation, USA) in Experiment A and the diets containing fish oil (Tuna RoPUFA, Nu-Mega, Australia) in Experiment B was equal and moderate, namely 0.2 DHA g/100 g. Average daily intake of the DHA per body weight was calculated based on the respective measured average body weight and food intake and was approximately 125 mg DHA/kg BW/day (Experiment A) and 110 mg DHA/kg BW/day (Experiment B). Average daily intake of the EPA per body weight was approximately 31 mg EPA/kg BW/day (Experiment A) and 27 mg EPA/kg BW/day (Experiment B). A detailed overview of the contents of diets is presented in Table 1. Diets were manufactured by Research Diet Services, Wijk bij Duurstede, The Netherlands (Experiment A) and Ssniff Spezialdiäten, Soest, Germany (Experiment B) and were stored at −20 °C until use, in order to prevent oxidation of lipids. Analysis of the diets confirmed the calculated fatty acid composition and confirmed that the control diets and the lecithin diet did not contain DHA or EPA.

**Table 1:** Detailed compositions of the experimental diets used in Experiment A and Experiment B

Ingredients (g/100 g diet)	Diets Experiment A			
	Control	Lecithin	Vegetable DHA oil	Vegetable DHA oil + lecithin
Wheat	25.87	25.87	25.87	25.87
Barley	25.00	25.00	25.00	25.00
Semolina	25.00	24.60	25.00	24.60
Soybean meal	8.50	8.50	8.50	8.50
Bentonite	1.00	1.00	1.00	1.00
Whey powder	5.00	5.00	5.00	5.00
Soy oil	1.90	1.90	1.76	1.76
Corn oil	2.20	2.20	1.90	1.90
Coconut fat	0.90	0.90	0.80	0.80
Vegetable algae DHA oil (DHASCO oil)	–	–	0.54	0.54
Vitamin/mineral mix (including choline)	2.20	2.20	2.20	2.20
CaCO_3_	1.40	1.40	1.40	1.40
Dicalcium phosphate	0.30	0.30	0.30	0.30
NaCl	0.50	0.50	0.50	0.50
l-Lysine HCl	0.18	0.18	0.18	0.18
dl-Methionine	0.05	0.05	0.05	0.05
Soy lecithin (Emulpur IP)	–	0.40	–	0.40
Sum	100.00	100.00	100.00	100.00

Ingredients (g/100 g diet)	Diets Experiment B		
	Control	Fish oil	Fish oil + lecithin
Cornstarch, pre-gelatinized	34.59	34.59	34.34
Maltodextrin, 10 DE	15.50	15.50	15.50
Sucrose	10.00	10.00	10.00
Dextrose	10.00	10.00	10.00
Cellulose powder	5.00	5.00	5.00
Casein	14.00	14.00	14.00
Soy oil	3.00	3.00	2.25
Corn oil	2.10	1.50	1.50
Coconut fat	0.90	0.70	0.70
Fish oil (Tuna RoPUFA)	–	0.80	0.80
Mineral mix (AIN-93M-MX)	3.50	3.50	3.50
Vitamin mix (AIN-93-VX)	1.00	1.00	1.00
Choline chloride (50 %)	0.23	0.23	0.23
l-Cystine	0.18	0.18	0.18
Tert-butylhydroquinone	0.0008	0.0008	0.0008
Soy lecithin (Emulpur IP)	–	–	1.00
Sum	100.00	100.00	100.00

### Experimental Design

In both experiments, all rats were provided with the respective control diet for 2 weeks before starting the intervention. Subsequently, animals were divided into the experimental groups that were matched according to their body weight and food intake and were placed on one of the experimental diets for 2 weeks (Experiment A) or 3 weeks (Experiment B).

### Sample Preparation

After the supplementation period, animals were food-deprived for 3–4 h and euthanized by inhalation of isoflurane and subsequent decapitation by guillotine. Trunk blood was collected through a funnel into EDTA-containing tubes. After centrifugation at 1300×*g* at 20 °C for 10 min (Experiment A) or 1750×*g* at 4 °C for 10 min (Experiment B), plasma and RBC (Experiment B only) were collected and analyzed for plasma fatty acid composition (Experiments A and B) and for RBC fatty acid composition and free choline (Experiment B only). Plasma sample preparation in Experiment A was not adequate for choline analysis as plasma free choline is not stable at room temperature and therefore these results are lacking. Based on the results of Experiment A, RBC samples were also collected in Experiment B.

### Plasma Fatty Acid Composition and Plasma Free Choline Analyses

Plasma and RBC total lipid fatty acid composition was detected using gas chromatography (GC). Total lipids were extracted from plasma and RBC using a modified Bligh and Dyer protocol [16] by adding methanol, 1 % EDTA solution, and dichloromethane to 100 µL plasma or approximately 150 µL RBC (non-quantitative). After vortexing for 5 min, samples were centrifuged at 1750×*g* for 10 min and the organic phase (dichloromethane and lipids) was collected. The dichloromethane layer was dried using a SpeedVac^®^ concentrator. Next, 2.0 mL methanol and 40 μL concentrated sulfuric acid (2 v/v %) were added to the dried extract and samples were heated at 100 °C for 60 min [17]. After cooling, 2 mL hexane and 0.5 mL 2.5 mol/L sodium hydroxide solution were added. Samples were subsequently vortexed and centrifuged for 5 min at 1750×*g* after which the upper layer was collected and dried using a SpeedVac^®^. Dried samples were subsequently dissolved in 125 μL iso-octane and analyzed by GC (Shimadzu Corporation, Kyoto, Japan) using flame ionization detection with a CP-SIL88 column (50 m × 0.25 mm id. 0.20 μm film thickness; Agilent Technologies, Inc., Santa Clara, CA, USA). The carrier gas was hydrogen and the make-up gas was nitrogen. GC oven temperature settings were as follows: initial temperature 150 °C for 3.75 min; temperature increment to 220 °C at 22 °C/min; and subsequent constant temperature of 220 °C for 14.07 min (in-house settings). Fatty acids were identified based on retention time using an external reference standard (GLC-461, Nu-Chek Prep, Inc., Elysian, MN, USA). Peak area was used as a measure of relative percent (mol%). An internal standard (1,2-dinonadecanoyl-*sn*-glycero-3-phosphocholine, Avanti Polar Lipids, Inc., AL, USA) was used for absolute quantification of fatty acids (µmol/L) in plasma. RBC fatty acids were not quantitatively pipetted and were therefore not absolutely quantified.

High performance liquid chromatography (HPLC) coupled to electrochemical detection of plasma free choline was performed using a commercially available choline assay HPLC method from Thermo Fisher Scientific (former ESA Biosciences, Chelmsford, MA, USA). Briefly, after protein precipitation, samples were centrifuged to remove proteins. The supernatant was injected into the HPLC (Thermo Fisher Scientific, former ESA Biosciences, Chelmsford, MA, USA) using a post-column immobilized enzyme reactor, in an on-line enzyme reaction to produce H_2_O_2_, which was detected electrochemically.

### Statistical Analyses

All statistical analyses were performed using SPSS (version 19, SPSS Inc., Chicago, IL, USA). Data were expressed as means ± SEM. *P* values <0.05 were considered significant. Effects of dietary treatments on body weight and food intake were analyzed using repeated-measures ANOVA with Diet (Experiment A) or dietary DHA and dietary lecithin (Experiment B) as the between-subject factor(s) and day as the within-subject factor. Plasma and RBC fatty acids relative and quantitative levels (DHA, EPA, total n-3 PUFA and n-6 PUFA) and choline concentrations were compared between rats fed the different experimental diets using two-way ANOVA with dietary DHA and dietary lecithin as between-subject factors (Experiment A) or one-way ANOVA (Experiment B). Post hoc comparisons were performed where appropriate.

## Results

### Experiment A

**Fig. 1:**
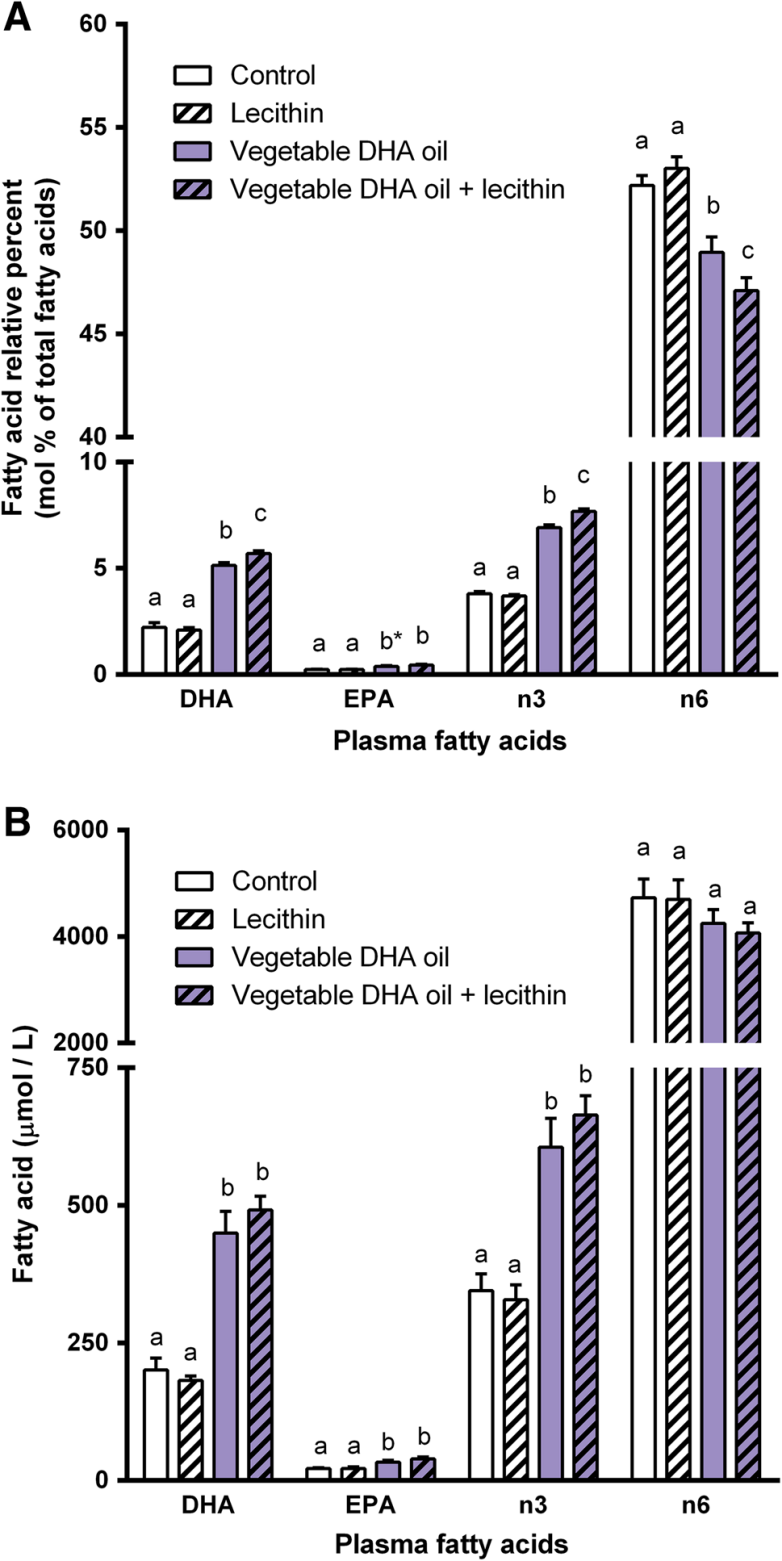
Experiment A: effects of dietary supplementation of vegetable DHA oil, lecithin or the combination of both on **a** relative percent levels (mol%) and **b** quantitative concentrations (µmol/L) of DHA, EPA, total n-3 PUFA, and total n-6 PUFA in plasma of rats. Plasma DHA, EPA, total n-3 PUFA, and total n-6 PUFA levels in rats that received one of the four experimental diets (control, lecithin, vegetable DHA oil, vegetable DHA oil + lecithin) for 2 weeks. Values are means, with the SEM represented by *vertical bars*. *Different letters* indicate mean values were significantly different (*P* < 0.05). *P = 0.077 *vs.* vegetable DHA oil + lecithin. *n* = 6 per experimental group

After feeding all animals the control diet for 2 weeks, they were divided into four experimental groups that were matched according to their body weight and food intake. During the supplementation period of 2 weeks body weight was neither affected by dietary DHA supplementation (*P* = 0.55) nor by dietary lecithin supplementation (*P* = 0.72). Also no effects on food intake were found (dietary DHA: *P* = 0.89); dietary lecithin: *P* = 0.99). Dietary DHA supplementation increased relative plasma levels of DHA (*P* < 0.001), EPA (*P* < 0.001), and total n-3 PUFA (*P* < 0.001), and decreased total n-6 PUFA (*P* < 0.001). Plasma levels of DHA (*P* = 0.20), EPA (*P* = 0.21), and total n-6 PUFA (*P* = 0.41) were not affected by dietary lecithin supplementation, whereas plasma total n-3 PUFA level (*P* = 0.007) was affected. Furthermore, interactions between dietary DHA and lecithin supplementation were found for plasma DHA (*P* = 0.039), total n-3 PUFA (*P* = 0.001), and total n-6 PUFA (*P* = 0.041), however not for EPA (*P* = 0.19). These main and interaction effects indicate that combined dietary supplementation of DHA oil and lecithin increased the changes induced by DHA supplementation alone (Fig. 1a). Quantitative plasma concentrations of DHA (*P* < 0.001), EPA (*P* < 0.001), and total n-3 PUFA (*P* < 0.001) were also affected by dietary DHA supplementation, whereas total n-6 PUFA (*P* = 0.078) was not. No effects of dietary lecithin or interactions between dietary DHA and lecithin were found when expressing the plasma fatty acid levels as quantitative concentrations, although trends were apparent (Fig. 1b). Tables 2 and 3 give a complete overview of all plasma fatty acids measured, expressed as relative percent levels (Table 2) and quantitative concentrations (Table 3).

**Table 2:** Experiment A: effects of dietary supplementation of vegetable DHA oil, lecithin or the combination of both on plasma fatty acid composition (mol%) in rats

Plasma fatty acids (mol% of total fatty acids)	Experiment A			
	Dietary intervention			
	Control	Lecithin	Vegetable DHA oil	Vegetable DHA oil + lecithin
6:0	0.0 ± 0.0	0.0 ± 0.0	0.0 ± 0.0	0.0 ± 0.0
8:0	0.0 ± 0.0	0.0 ± 0.0	0.0 ± 0.0	0.0 ± 0.0
10:0	0.0 ± 0.0	0.0 ± 0.0	0.0 ± 0.0	0.0 ± 0.0
12:0	0.6 ± 0.2	0.7 ± 0.1	0.7 ± 0.1	0.6 ± 0.1
14:0	0.9 ± 0.1	0.9 ± 0.1	1.0 ± 0.1	1.0 ± 0.1
15:0	0.3 ± 0.0	0.3 ± 0.0	0.3 ± 0.0	0.3 ± 0.0
16:0	18.6 ± 0.2	18.6 ± 0.2	19.1 ± 0.3	19.8 ± 0.2
17:0	0.3 ± 0.0	0.3 ± 0.0	0.3 ± 0.0	0.3 ± 0.0
18:0	7.8 ± 0.4	7.6 ± 0.5	7.4 ± 0.4	7.1 ± 0.3
20:0	0.1 ± 0.0	0.1 ± 0.0	0.1 ± 0.0	0.1 ± 0.0
22:0	0.2 ± 0.0	0.2 ± 0.0	0.2 ± 0.0	0.2 ± 0.0
23:0	0.0 ± 0.0	0.0 ± 0.0	0.0 ± 0.0	0.0 ± 0.0
24:0	0.2 ± 0.0	0.2 ± 0.0	0.2 ± 0.0	0.2 ± 0.0
16:1n-7	1.0 ± 0.1	0.8 ± 0.1	1.1 ± 0.1	1.3 ± 0.2
18:1n-7	2.2 ± 0.1	2.0 ± 0.1	2.1 ± 0.2	2.3 ± 0.2
18:1n-9	11.2 ± 0.5	10.9 ± 0.5	11.1 ± 0.5	11.5 ± 0.4
20:1n-9	0.3 ± 0.0	0.3 ± 0.0	0.3 ± 0.0	0.3 ± 0.0
22:1n-9	0.0 ± 0.0	0.0 ± 0.0	0.0 ± 0.0	0.0 ± 0.0
24:1n-9	0.1 ± 0.0	0.1 ± 0.0	0.1 ± 0.0	0.1 ± 0.0
20:3n-9	0.1 ± 0.0	0.0 ± 0.0	0.0 ± 0.0	0.0 ± 0.0
18:2n-6	26.5 ± 1.4	28.3 ± 1.0	27.6 ± 0.7	28.1 ± 0.8
18:3n-6	0.4 ± 0.0	0.3 ± 0.0	0.2 ± 0.0	0.2 ± 0.0
20:2n-6	0.6 ± 0.0	0.6 ± 0.0	0.5 ± 0.0	0.6 ± 0.0
20:3n-6	0.6 ± 0.1	0.6 ± 0.0	0.7 ± 0.0	0.8 ± 0.0
20:4n-6	22.4 ± 1.5	21.8 ± 1.5	19.1 ± 1.3	16.6 ± 1.1
22:4n-6	1.1 ± 0.1	1.0 ± 0.1	0.5 ± 0.0	0.6 ± 0.0
22:5n-6	0.6 ± 0.1	0.4 ± 0.0	0.2 ± 0.0	0.2 ± 0.0
24:2n-6	0.0 ± 0.0	0.0 ± 0.0	0.0 ± 0.0	0.0 ± 0.0
18:3n-3	0.7 ± 0.1	0.8 ± 0.1	0.9 ± 0.1	0.9 ± 0.1
18:4n-3	0.0 ± 0.0	0.0 ± 0.0	0.0 ± 0.0	0.0 ± 0.0
20:5n-3	0.2 ± 0.0	0.2 ± 0.0	0.4 ± 0.0	0.5 ± 0.0
22:5n-3	0.6 ± 0.1	0.5 ± 0.0	0.5 ± 0.0	0.6 ± 0.0
22:6n-3	2.2 ± 0.2	2.1 ± 0.1	5.1 ± 0.1	5.7 ± 0.1
Total SFA	29.1 ± 0.2	29.1 ± 0.1	29.4 ± 0.3	29.6 ± 0.2
Total MUFA	14.8 ± 0.5	14.2 ± 0.7	14.7 ± 0.8	15.5 ± 0.6
Total PUFA	56.1 ± 0.5	56.7 ± 0.6	55.9 ± 0.7	54.8 ± 0.6
Total n-3 PUFA	3.8 ± 0.1	3.7 ± 0.1	6.9 ± 0.1	7.7 ± 0.1
Total n-6 PUFA	52.2 ± 0.5	53.0 ± 0.6	49.0 ± 0.7	47.1 ± 0.6
Total fatty acids	100.0	100.0	100.0	100.0

Values are means ± SEM

**Table 3:** Experiment A: effects of dietary supplementation of vegetable DHA oil, lecithin or the combination of both on plasma fatty acid quantitative concentrations (µmol/L) in rats

Plasma fatty acids (µmol/L)	Experiment A			
	Dietary intervention			
	Control	Lecithin	Vegetable DHA oil	Vegetable DHA oil + lecithin
6:0	0.0 ± 0.0	0.0 ± 0.0	0.0 ± 0.0	0.0 ± 0.0
8:0	0.0 ± 0.0	0.0 ± 0.0	0.0 ± 0.0	0.0 ± 0.0
10:0	2.3 ± 1.2	2.4 ± 0.9	2.7 ± 0.6	1.9 ± 0.6
12:0	57.9 ± 18.5	70.2 ± 18.6	65.4 ± 10.8	51.6 ± 6.6
14:0	80.5 ± 15.3	80.9 ± 14.2	88.0 ± 10.9	86.9 ± 7.0
15:0	27.0 ± 2.5	29.7 ± 3.0	25.8 ± 2.2	26.8 ± 1.3
16:0	1700.2 ± 160.4	1658.4 ± 152.8	1669.9 ± 130.7	1711.4 ± 92.3
17:0	28.2 ± 1.5	29.3 ± 1.1	26.6 ± 1.6	26.3 ± 1.4
18:0	698.2 ± 35.0	661.1 ± 17.6	639.6 ± 32.0	615.4 ± 38.9
20:0	9.6 ± 1.2	10.2 ± 1.4	9.4 ± 0.8	8.5 ± 0.4
22:0	18.1 ± 1.7	17.6 ± 1.8	14.1 ± 1.1	14.5 ± 0.4
23:0	3.2 ± 0.8	3.2 ± 0.5	2.9 ± 0.5	3.1 ± 0.2
24:0	18.3 ± 1.2	19.5 ± 2.3	17.4 ± 1.0	17.4 ± 1.0
16:1n-7	91.5 ± 14.0	72.3 ± 12.5	95.7 ± 17.9	110.2 ± 17.5
18:1n-7	205.2 ± 21.5	184.1 ± 22.1	189.7 ± 27.5	201.4 ± 17.6
18:1n-9	1031.2 ± 132.8	984.0 ± 125.9	981.3 ± 114.0	989.7 ± 51.5
20:1n-9	26.7 ± 3.7	27.6 ± 4.2	25.9 ± 3.7	24.5 ± 1.0
22:1n-9	2.3 ± 0.5	2.5 ± 0.5	2.1 ± 0.3	2.2 ± 0.3
24:1n-9	11.0 ± 0.4	12.1 ± 1.3	11.1 ± 0.7	12.2 ± 0.7
20:3n-9	6.7 ± 3.3	2.6 ± 0.4	2.8 ± 1.3	2.8 ± 0.6
18:2n-6	2427.3 ± 292.0	2551.9 ± 303.6	2419.2 ± 202.0	2424.5 ± 126.3
18:3n-6	33.6 ± 3.8	27.9 ± 3.0	21.0 ± 2.3	20.4 ± 2.4
20:2n-6	50.6 ± 5.2	51.6 ± 4.0	44.9 ± 5.1	50.0 ± 2.4
20:3n-6	58.5 ± 7.0	56.4 ± 7.3	61.3 ± 6.4	70.4 ± 3.4
20:4n-6	1996.4 ± 110.1	1887.3 ± 74.0	1635.1 ± 72.7	1433.5 ± 116.6
22:4n-6	102.1 ± 12.6	84.5 ± 5.5	48.3 ± 6.0	50.7 ± 2.6
22:5n-6	57.9 ± 11.9	36.6 ± 1.6	16.0 ± 2.9	16.9 ± 1.0
24:2n-6	3.0 ± 0.5	2.9 ± 0.4	2.2 ± 0.3	1.9 ± 0.2
18:3n-3	69.1 ± 13.4	76.1 ± 14.4	77.4 ± 10.1	76.8 ± 6.4
18:4n-3	2.8 ± 1.0	1.5 ± 0.9	1.3 ± 0.9	0.2 ± 0.2
20:5n-3	21.7 ± 1.6	21.7 ± 3.0	33.5 ± 3.4	39.2 ± 3.7
22:5n-3	50.7 ± 5.0	47.2 ± 3.3	43.6 ± 3.4	56.1 ± 5.0
22:6n-3	200.8 ± 21.6	181.8 ± 7.8	449.8 ± 39.1	491.7 ± 25.0
Total SFA	2643.4 ± 223.7	2582.5 ± 211.7	2561.9 ± 180.8	2563.8 ± 132.2
Total MUFA	1367.9 ± 169.1	1282.6 ± 164.0	1305.9 ± 161.6	1340.2 ± 72.6
Total PUFA	5081.3 ± 380.6	5029.9 ± 392.7	4856.2 ± 308.3	4735.0 ± 220.5
Total n-3 PUFA	345.1 ± 30.2	328.3 ± 27.2	605.5 ± 52.6	664.0 ± 35.3
Total n-6 PUFA	4729.4 ± 352.0	4699.0 ± 365.4	4247.9 ± 256.8	4068.2 ± 188.2
Total fatty acids	9092.6 ± 771.1	8895.0 ± 765.0	8724.0 ± 647.0	8639.0 ± 399.5

Values are means ± SEM

### Experiment B

**Fig. 2:**
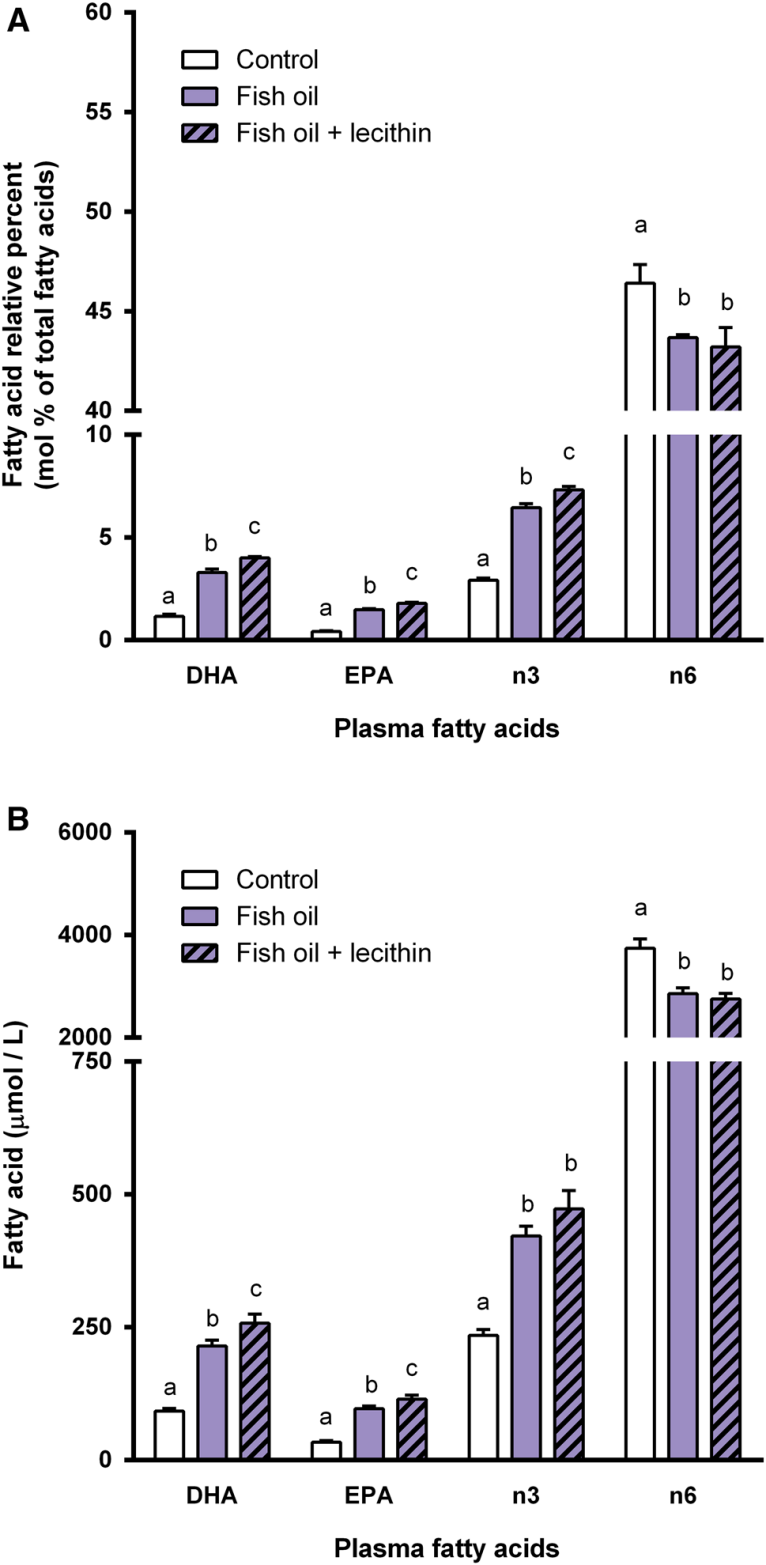
Experiment B: effects of dietary supplementation of fish oil or combined dietary supplementation of fish oil and lecithin on **a** relative percent levels (mol%) and **b** quantitative concentrations (µmol/L) of DHA, EPA, total n-3 PUFA, and total n-6 PUFA in plasma of rats. Plasma DHA, EPA, total n-3 PUFA, and total n-6 PUFA levels in rats that received one of the three experimental diets (control, fish oil, fish oil + lecithin) for 3 weeks. Values are means, with the SEM represented by *vertical bars*. *Different letters* indicate mean values were significantly different (*P* < 0.025). *n* = 7–8 per experimental group, 1 outlier was omitted from the data

Animals were divided into three experimental groups that were matched according to their body weights and food intake after feeding the animals the control diet for 2 weeks. Body weight (*P* = 0.78) and food intake (*P* = 0.47) did not differ between the experimental groups over the course of the 3-week intervention period. Relative plasma levels of DHA (*P* < 0.001), EPA (*P* < 0.001), total n-3 PUFA (*P* < 0.001), and total n-6 PUFA (*P* = 0.023) were affected by the experimental diets (Fig. 2a). Animals fed the fish oil diet displayed increased plasma levels of DHA (*P* < 0.001), EPA (*P* < 0.001), and total n-3 PUFA (*P* < 0.001), and a decreased plasma total n-6 PUFA level (*P* = 0.031) as compared to the control diet. The increases in plasma DHA, EPA, total n-3 PUFA were augmented by additional supplementation with lecithin, i.e., plasma DHA (*P* < 0.001), EPA (*P* < 0.001), and total n-3 PUFA (*P* = 0.001) were higher in rats fed the fish oil + lecithin diet as compared to those fed the fish oil diet. Expressing the plasma fatty acid levels as quantitative concentrations revealed similar effects of the fish oil diet and the fish-oil + lecithin diet. Plasma concentrations of DHA (*P* < 0.001), EPA (*P* < 0.001), total n-3 PUFA (*P* < 0.001), and total n-6 PUFA (*P* < 0.001) differed between rats fed one of the three experimental diets (Fig. 2b). Compared to animals fed the control diet or the Fish oil diet, those fed the fish oil + lecithin diet displayed increased plasma concentrations of DHA (*P* < 0.001 *vs.* control diet; *P* = 0.022 *vs.* fish oil diet) and EPA (*P* < 0.001 *vs.* control diet; *P* = 0.028 *vs.* fish oil diet). Tables 4 and 5 give a complete overview of all plasma fatty acids measured, expressed as relative percent levels (Table 4) and quantitative concentrations (Table 5).

**Table 4:** Experiment B: effects of dietary supplementation of fish oil or combined dietary supplementation of fish oil and lecithin on plasma fatty acid composition (mol%) in rats

Plasma fatty acids (mol% of total fatty acids)	Experiment B		
	Dietary intervention		
	Control	Fish oil	Fish oil + lecithin
6:0	0.0 ± 0.0	0.0 ± 0.0	0.0 ± 0.0
8:0	0.0 ± 0.0	0.0 ± 0.0	0.0 ± 0.0
10:0	0.0 ± 0.0	0.0 ± 0.0	0.0 ± 0.0
12:0	0.4 ± 0.1	0.2 ± 0.0	0.2 ± 0.0
14:0	1.2 ± 0.1	0.9 ± 0.0	1.0 ± 0.1
15:0	0.2 ± 0.0	0.2 ± 0.0	0.3 ± 0.0
16:0	21.2 ± 0.2	22.4 ± 0.2	22.9 ± 0.3
17:0	0.1 ± 0.0	0.1 ± 0.0	0.2 ± 0.0
18:0	6.8 ± 0.4	7.2 ± 0.2	7.2 ± 0.3
20:0	0.1 ± 0.0	0.1 ± 0.0	0.1 ± 0.0
22:0	0.2 ± 0.0	0.2 ± 0.0	0.2 ± 0.0
23:0	0.0 ± 0.0	0.0 ± 0.0	0.0 ± 0.0
24:0	0.3 ± 0.0	0.3 ± 0.0	0.3 ± 0.0
16:1n-7	2.8 ± 0.3	2.4 ± 0.1	2.4 ± 0.3
18:1n-7	3.5 ± 0.2	2.9 ± 0.1	2.7 ± 0.1
18:1n-9	13.3 ± 0.7	12.5 ± 0.2	11.6 ± 0.5
20:1n-9	0.2 ± 0.0	0.1 ± 0.0	0.1 ± 0.0
22:1n-9	0.0 ± 0.0	0.0 ± 0.0	0.0 ± 0.0
24:1n-9	0.1 ± 0.0	0.1 ± 0.0	0.2 ± 0.0
20:3n-9	0.2 ± 0.0	0.1 ± 0.0	0.2 ± 0.0
18:2n-6	26.4 ± 0.7	27.4 ± 0.5	27.6 ± 0.4
18:3n-6	0.4 ± 0.0	0.2 ± 0.0	0.2 ± 0.0
20:2n-6	0.3 ± 0.0	0.3 ± 0.0	0.2 ± 0.0
20:3n-6	0.4 ± 0.0	0.5 ± 0.0	0.5 ± 0.0
20:4n-6	18.4 ± 1.1	14.8 ± 0.5	14.3 ± 0.8
22:4n-6	0.3 ± 0.1	0.4 ± 0.1	0.3 ± 0.1
22:5n-6	0.1 ± 0.0	0.1 ± 0.0	0.1 ± 0.0
24:2n-6	0.0 ± 0.0	0.0 ± 0.0	0.0 ± 0.0
18:3n-3	0.9 ± 0.0	1.0 ± 0.0	0.8 ± 0.1
18:4n-3	0.0 ± 0.0	0.0 ± 0.0	0.0 ± 0.0
20:5n-3	0.4 ± 0.0	1.5 ± 0.1	1.8 ± 0.1
22:5n-3	0.5 ± 0.0	0.7 ± 0.0	0.8 ± 0.0
22:6n-3	1.2 ± 0.1	3.3 ± 0.2	4.0 ± 0.1
Total SFA	30.6 ± 0.3	31.7 ± 0.3	32.4 ± 0.3
Total MUFA	19.8 ± 1.1	18.0 ± 0.3	16.9 ± 0.9
Total PUFA	49.5 ± 1.0	50.3 ± 0.2	50.7 ± 0.9
Total n-3 PUFA	2.9 ± 0.1	6.5 ± 0.2	7.3 ± 0.2
Total n-6 PUFA	46.4 ± 0.9	43.7 ± 0.1	43.2 ± 1.0
Total fatty acids	100.0	100.0	100.0

Values are means ± SEM

**Table 5:** Experiment B: effects of dietary supplementation of fish oil or combined dietary supplementation of fish oil and lecithin on plasma fatty acid quantitative concentrations (µmol/L) in rats

Plasma fatty acids (µmol/L)	Experiment B		
	Dietary intervention		
	Control	Fish oil	Fish oil + lecithin
6:0	0.0 ± 0.0	0.0 ± 0.0	0.0 ± 0.0
8:0	0.0 ± 0.0	0.0 ± 0.0	0.0 ± 0.0
10:0	0.0 ± 0.0	0.0 ± 0.0	0.0 ± 0.0
12:0	40.3 ± 13.8	14.9 ± 2.3	12.9 ± 2.8
14:0	104.2 ± 19.2	61.3 ± 4.5	62.7 ± 7.5
15:0	15.0 ± 1.1	16.0 ± 0.9	17.0 ± 1.4
16:0	1726.0 ± 111.3	1462.0 ± 50.1	1471.8 ± 81.1
17:0	9.0 ± 1.3	9.7 ± 2.2	11.6 ± 1.3
18:0	544.7 ± 26.4	470.0 ± 18.4	456.3 ± 13.8
20:0	6.9 ± 0.7	6.3 ± 0.4	6.7 ± 0.6
22:0	18.5 ± 1.1	14.1 ± 1.0	14.8 ± 1.0
23:0	0.0 ± 0.0	0.0 ± 0.0	0.0 ± 0.0
24:0	22.2 ± 0.7	18.5 ± 0.8	21.5 ± 1.2
16:1n-7	230.3 ± 36.0	155.7 ± 7.8	158.2 ± 23.6
18:1n-7	282.9 ± 29.9	187.8 ± 7.7	176.0 ± 16.3
18:1n-9	1101.0 ± 124.7	815.0 ± 38.0	750.9 ± 68.1
20:1n-9	12.6 ± 1.9	8.3 ± 0.5	5.9 ± 1.1
22:1n-9	0.0 ± 0.0	0.0 ± 0.0	0.0 ± 0.0
24:1n-9	9.3 ± 0.5	9.5 ± 0.2	9.8 ± 0.8
20:3n-9	17.6 ± 1.3	9.3 ± 0.8	9.6 ± 0.9
18:2n-6	2151.2 ± 165.7	1793.2 ± 85.1	1768.8 ± 96.2
18:3n-6	29.6 ± 1.2	16.0 ± 1.6	14.0 ± 1.3
20:2n-6	27.4 ± 2.4	17.4 ± 1.4	15.9 ± 1.3
20:3n-6	35.8 ± 2.2	34.2 ± 1.7	33.1 ± 2.1
20:4n-6	1463.9 ± 45.1	963.2 ± 45.5	902.7 ± 16.3
22:4n-6	28.1 ± 9.0	26.9 ± 10.4	17.0 ± 6.9
22:5n-6	9.1 ± 2.9	6.6 ± 0.4	3.7 ± 1.4
24:2n-6	0.0 ± 0.0	0.0 ± 0.0	0.0 ± 0.0
18:3n-3	71.9 ± 8.5	63.7 ± 3.9	50.6 ± 8.4
18:4n-3	0.0 ± 0.0	0.0 ± 0.0	0.0 ± 0.0
20:5n-3	33.8 ± 2.7	96.6 ± 5.0	115.0 ± 7.3
22:5n-3	37.1 ± 2.0	46.7 ± 2.8	49.2 ± 5.3
22:6n-3	92.1 ± 5.0	214.5 ± 11.2	257.7 ± 16.9
Total SFA	2486.9 ± 156.9	2072.6 ± 66.0	2075.2 ± 96.7
Total MUFA	1636.1 ± 187.7	1176.4 ± 52.0	1100.9 ± 107.6
Total PUFA	3997.7 ± 189.1	3288.2 ± 130.7	3237.4 ± 139.2
Total n-3 PUFA	234.9 ± 10.9	421.5 ± 18.7	472.5 ± 35.0
Total n-6 PUFA	3745.2 ± 178.2	2857.4 ± 115.9	2755.2 ± 106.6
Total fatty acids	8120.6 ± 510.0	6537.3 ± 243.6	6413.5 ± 327.5

Values are means ± SEM

**Fig. 3:**
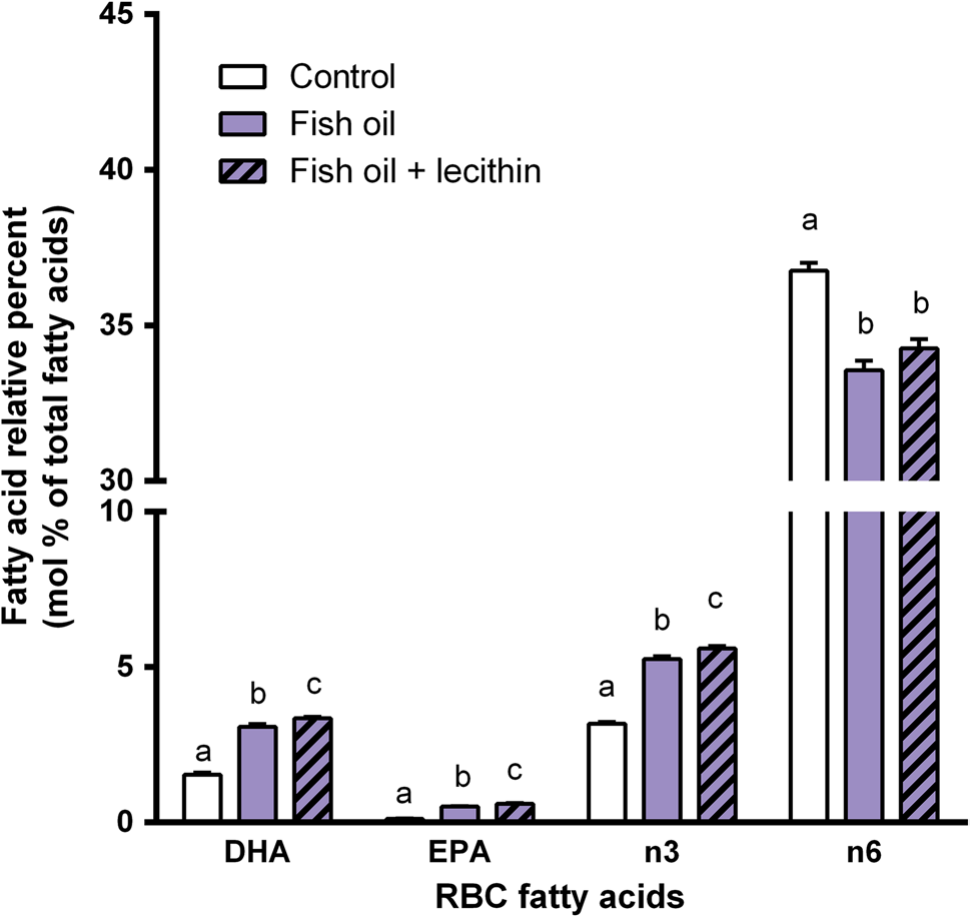
Experiment B: effects of dietary supplementation of fish oil or combined dietary supplementation of fish oil and lecithin on relative percent levels (mol%) of DHA, EPA, total n-3 PUFA, and total n-6 PUFA in RBC of rats. RBC DHA, EPA, total n-3 PUFA, and total n-6 PUFA levels in rats that received one of the three experimental diets (control, fish oil, fish oil + lecithin) for 3 weeks. Values are means, with the SEM represented by *vertical bars*. *Different letters* indicate mean values were significantly different (*P* < 0.025). *n* = 8 per experimental group

In RBC, similar differences in levels of DHA, EPA, n-3 PUFA, and n-6 PUFA between the experimental groups were found as obtained in plasma. Relative RBC levels of DHA (*P* < 0.001), EPA (*P* < 0.001), total n-3 PUFA (*P* < 0.001), and total n-6 PUFA (*P* < 0.001) also were affected by the experimental diets (Fig. 3). Compared to animals fed the control diet or the fish oil diet, those fed the fish oil + lecithin diet displayed increased RBC levels of DHA (*P* < 0.001 *vs.* control diet; *P* = 0.009 *vs.* fish oil diet), EPA (*P* < 0.001 *vs.* control diet; *P* = 0.006 *vs.* fish oil diet), and total n-3 PUFA (*P* < 0.001 *vs.* control diet; *P* = 0.004 *vs.* fish oil diet). Table 6 gives a complete overview of all RBC fatty acids measured, expressed as relative percent levels.

**Table 6:** Experiment B: effects of dietary supplementation of fish oil or combined dietary supplementation of fish oil and lecithin on RBC fatty acid composition (mol%) in rats

RBC fatty acids (mol% of total fatty acids)	Experiment B		
	Dietary intervention		
	Control	Fish oil	Fish oil + lecithin
6:0	0.0 ± 0.0	0.0 ± 0.0	0.0 ± 0.0
8:0	0.0 ± 0.0	0.0 ± 0.0	0.0 ± 0.0
10:0	0.0 ± 0.0	0.0 ± 0.0	0.0 ± 0.0
12:0	0.1 ± 0.0	0.1 ± 0.0	0.1 ± 0.0
14:0	0.6 ± 0.0	0.5 ± 0.0	0.5 ± 0.0
15:0	0.2 ± 0.0	0.3 ± 0.0	0.3 ± 0.0
16:0	34.7 ± 0.3	36.4 ± 0.4	35.5 ± 0.3
17:0	0.3 ± 0.0	0.3 ± 0.0	0.4 ± 0.0
18:0	10.1 ± 0.3	10.1 ± 0.2	10.4 ± 0.2
20:0	0.1 ± 0.0	0.1 ± 0.0	0.1 ± 0.0
22:0	0.4 ± 0.0	0.4 ± 0.0	0.4 ± 0.0
23:0	0.0 ± 0.0	0.0 ± 0.0	0.0 ± 0.0
24:0	1.1 ± 0.0	1.0 ± 0.0	1.1 ± 0.0
16:1n-7	0.9 ± 0.1	0.8 ± 0.0	0.8 ± 0.1
18:1n-7	3.5 ± 0.1	3.2 ± 0.1	3.0 ± 0.1
18:1n-9	7.2 ± 0.2	7.0 ± 0.1	6.6 ± 0.2
20:1n-9	0.1 ± 0.0	0.1 ± 0.0	0.1 ± 0.0
22:1n-9	0.0 ± 0.0	0.0 ± 0.0	0.0 ± 0.0
24:1n-9	0.7 ± 0.0	0.7 ± 0.0	0.7 ± 0.0
20:3n-9	0.2 ± 0.0	0.1 ± 0.0	0.1 ± 0.0
18:2n-6	14.2 ± 0.5	14.5 ± 0.3	15.4 ± 0.3
18:3n-6	0.1 ± 0.0	0.1 ± 0.0	0.1 ± 0.0
20:2n-6	0.4 ± 0.0	0.4 ± 0.0	0.4 ± 0.0
20:3n-6	0.4 ± 0.0	0.5 ± 0.0	0.5 ± 0.0
20:4n-6	19.6 ± 0.3	16.6 ± 0.2	16.5 ± 0.3
22:4n-6	1.6 ± 0.0	1.2 ± 0.0	1.2 ± 0.0
22:5n-6	0.3 ± 0.0	0.2 ± 0.0	0.2 ± 0.0
24:2n-6	0.0 ± 0.0	0.0 ± 0.0	0.0 ± 0.0
18:3n-3	0.2 ± 0.0	0.3 ± 0.0	0.2 ± 0.0
18:4n-3	0.0 ± 0.0	0.0 ± 0.0	0.0 ± 0.0
20:5n-3	0.1 ± 0.0	0.5 ± 0.0	0.6 ± 0.0
22:5n-3	1.3 ± 0.0	1.4 ± 0.0	1.4 ± 0.0
22:6n-3	1.5 ± 0.1	3.1 ± 0.1	3.4 ± 0.0
Total SFA	47.5 ± 0.4	49.2 ± 0.3	48.7 ± 0.3
Total MUFA	12.5 ± 0.4	11.9 ± 0.2	11.3 ± 0.3
Total PUFA	40.1 ± 0.3	38.9 ± 0.3	40.0 ± 0.3
Total n-3 PUFA	3.2 ± 0.1	5.3 ± 0.1	5.6 ± 0.1
Total n-6 PUFA	36.8 ± 0.3	33.6 ± 0.3	34.3 ± 0.3
Total fatty acids	100.0	100.0	100.0

Values are means ± SEM

**Fig. 4:**
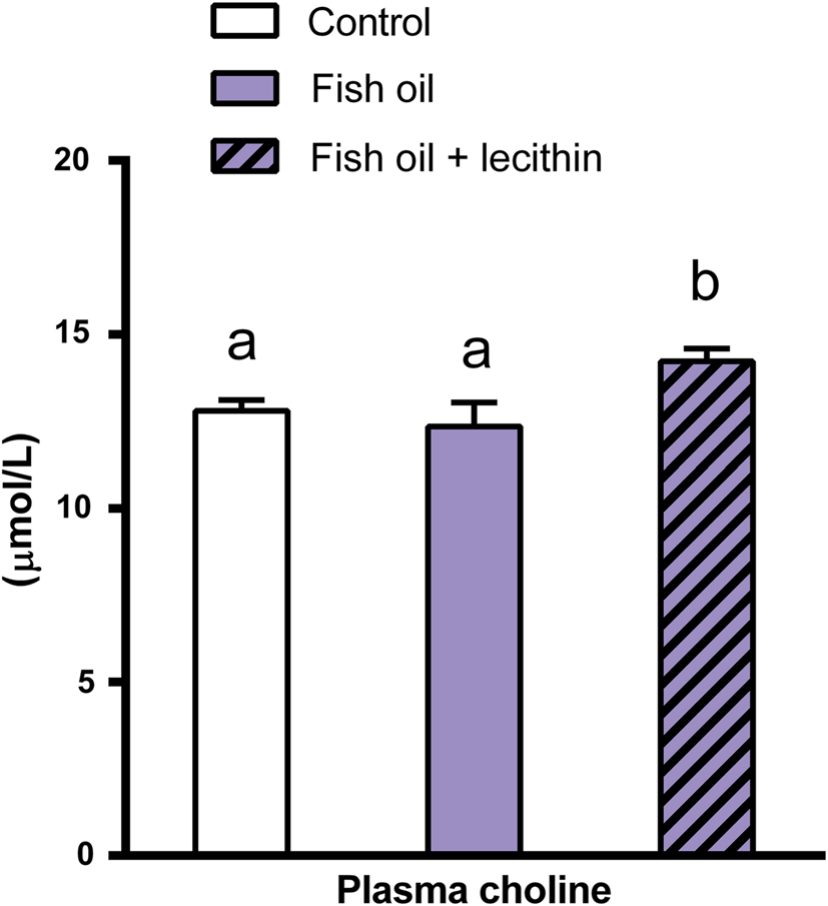
Experiment B: effects of dietary supplementation of fish oil or combined dietary supplementation of fish oil and lecithin on rat plasma free choline concentration (µmol/L). Plasma free choline concentration in rats that received one of the three experimental diets for 3 weeks (control, fish oil, fish oil + lecithin). Values are means, with the SEM represented by *vertical bars*. *Different letters* indicate mean values were significantly different (*P* < 0.05). *n* = 8 per experimental group

Plasma free choline concentration was also affected by the experimental diets (*P* = 0.030, Fig. 4). In animals receiving the lecithin-containing diet, plasma free choline concentration was higher as compared to those receiving the diets without lecithin (*P* = 0.049 *vs.* control diet; *P* = 0.012 *vs.* fish oil diet).

## Discussion

The present experiments indicate that dietary crude lecithin can increase the systemic availability of dietary DHA with combined intake. Dietary supplementation of lecithin, which itself does not contain any DHA, increased levels of DHA, EPA, and total n-3 PUFA in plasma and RBC, and decreased levels of total n-6 PUFA in plasma of rats fed DHA oil-containing diets. Thus, while dietary lecithin supplementation alone did not affect DHA levels in plasma and RBC, lecithin further increased the rise in DHA induced by dietary DHA supplementation (*vs.* control diets) with up to 61 % (relative levels) and 47 % (quantitative concentration) in plasma, and with 18 % (relative level) in RBC. Furthermore, dietary lecithin, being a source of PtdCho, increased plasma choline concentration with 11 %.

Dietary supplementation of moderate levels of DHA (i.e., 0.2 %), either as vegetable algae oil or as fish oil, affected relative and quantitative plasma levels and relative RBC fatty acid levels as expected. Previous studies, both experimental [4, 5, 18–21] and clinical [3, 22, 23], repeatedly demonstrated that plasma and RBC levels of DHA increase relatively fast in a dose-dependent manner after oral administration of DHA. In human, steady state plasma concentrations of DHA after supplementation is reached within 1 week up to 1 month, whereas for RBC this takes several months [3, 22]. In rats, fatty acid levels in plasma and RBC represents dietary intake of fats over a relatively short-term period of 1–4 weeks [24]. Hence, the plasma and RBC samples from the rats in the current study reflect the effects of the changes in dietary pattern (i.e., changes dietary DHA-containing oils and lecithin content) over the weeks of the applied supplementation period.

Dietary crude lecithin is a source of active compounds as it is digested into fatty acids, lysophospholipids, phosphatidic acid, glycerol, monoglycerides, and other compounds, including choline and ethanolamine. These digestion products are subsequently absorbed and further metabolized. For example, the intake of lecithin has been shown to increase serum levels of choline in humans [25] and rats [26]. In Experiment B, it was confirmed that lecithin supplementation increases plasma choline concentration in rats.

Systemic DHA is known to be transferred across the blood–brain barrier, as shown in both experimental [27, 28] and clinical studies [6, 29, 30]. In fact, dietary supplementation with DHA (in various formulas) has repeatedly been shown to affect membrane composition [5, 18, 31–39], thereby influencing its biophysical properties. A high level of n-3 PUFA in neuronal membranes is associated with favorable effects on numerous membrane-dependent processes and thus neuronal functioning [1, 2]. Likewise, small increases in plasma choline can exert significant effects on brain choline levels [40], and in fact, lecithin intake has been shown to increase both plasma and brain choline levels in the rat [26]. Brain choline levels in turn control the rates at which it is utilized to form acetylcholine [41, 42]. In addition, DHA, EPA and choline [43–45] are known precursors for phospholipid synthesis needed for membrane formation and integrity. By increasing the systemic availability of dietary DHA and by increasing the systemic levels of choline, dietary lecithin putatively leads to increased uptake of DHA and choline into the brain, with subsequent effects on neuronal membrane formation and function.

**Fig. 5:**
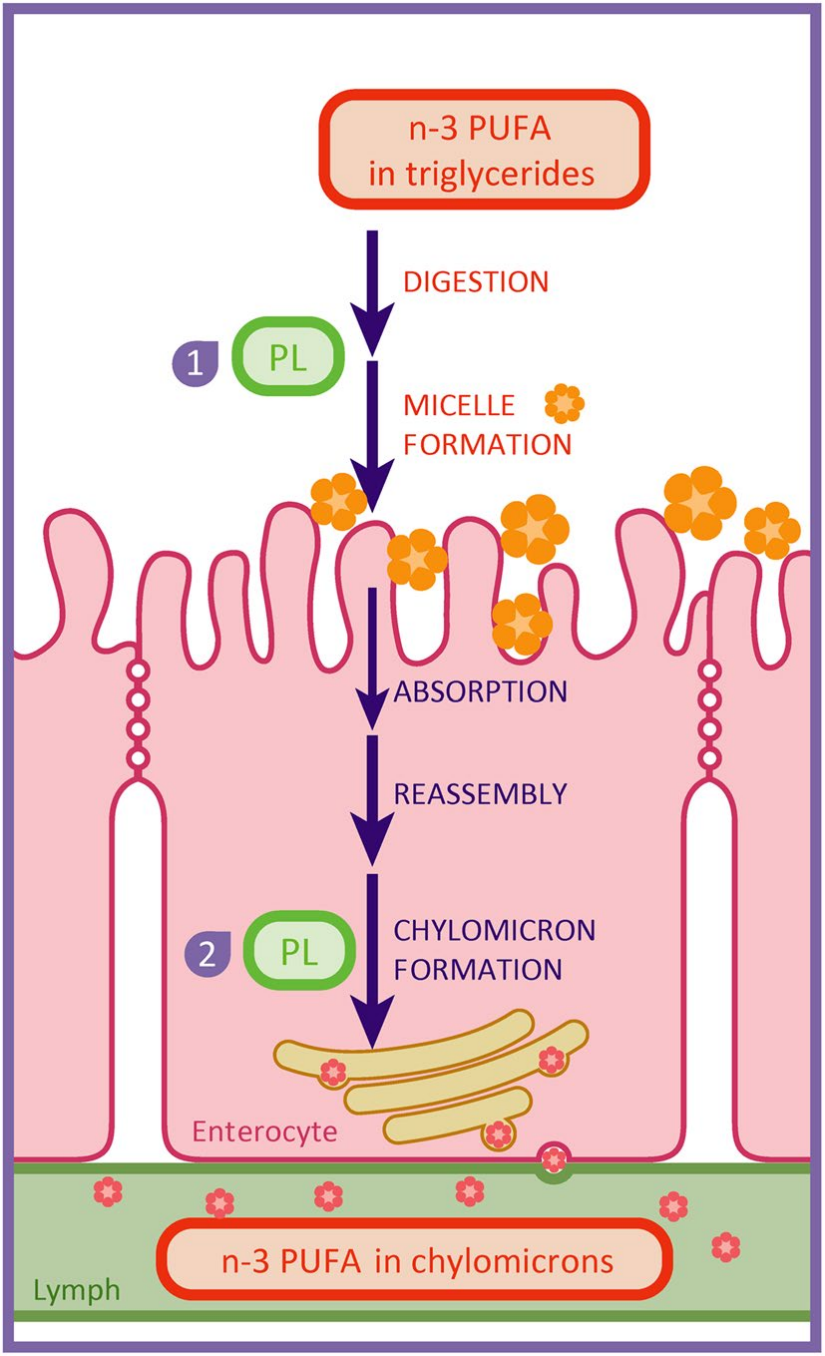
Schematic representation of the effects of dietary phospholipids on enhancing the absorption of dietary n-3 PUFA from the gut into the enterocytes and the lymph. Dietary phospholipids may increase the bioavailability of dietary n-3 PUFA by facilitating emulsification (*1*) and by increasing chylomicron formation (*2*). Figure is adapted from van Wijk *et al.* [54] with permission from IOS press. *PL* dietary phospholipids

The effects of lecithin supplementation on the systemic availability of dietary n-3 PUFA might be explained by an increased absorption from the gut into the enterocytes and the lymph. Dietary phospholipids could enhance the absorption of n-3 PUFA from the gut, since they are known to facilitate the emulsification of dietary fat in the lumen and subsequent micelle formation for absorption into the enterocytes [8, 46]. Moreover, dietary phospholipids may increase the intestinal uptake of fat by increasing the formation of chylomicrons in enterocytes and their secretion into the lymph [7, 10]. In the current study, the highest percentage increases in DHA were found in Experiment B in which a higher dose of crude lecithin was used (0.4 % in Experiment A and 1.0 % in Experiment B), possibly indicating dose-dependency. Figure 5 shows a schematic representation of these effects of dietary phospholipids.

Several investigators have previously attempted to demonstrate additive effects of lecithin or phospholipids or other emulsifiers on the bioavailability of triglycerides after oral intake in various models and using different approaches. Various studies thereby focused on the effects of pre-emulsification of oil with various emulsifiers, including lecithin [13–15, 47–49]. These studies distinctly show that single oral administration of pre-emulsified oil has a better bioavailability than non-emulsified oil. For example, Nishimukai *et al.* showed that duodenal infusion of an emulsion of triglycerides plus lecithin induces higher portal plasma triglycerides as compared to infusion of triglycerides alone [10]. In one study, the incorporation of radiolabeled DHA into various rat tissues were measured after a single dose of orally gavaged radiolabeled DHA, with or without administration of phospholipids (without emulsification), and showed no differential effects on the level of radiolabeled DHA in whole blood after 24 h [11]. Studies examining the effects of combined dietary supplementation of lecithin or phospholipids and (non-emulsified) triglycerides on systemic availability of triglycerides are scarcer. An experiment in fish (carps) demonstrated stimulated intestinal triglyceride uptake into plasma when dietary triglycerides were combined with dietary phospholipids [9]. In contrast, Lim *et al.* did not find increased amounts of DHA in plasma after combined dietary supplementation with DHA and phospholipids compared to supplementation with DHA alone [18]. However, in this study DHA content of the diet with both DHA and phospholipid was half of the DHA diet, which makes the diets’ efficacy impossible to compare. Other investigators have tried to show superiority of DHA-containing phospholipids (i.e., phospholipids with DHA as one of the fatty acid chains) over DHA-containing triglycerides to increase tissue levels of DHA in both preclinical and clinical experiments [11, 21, 50–53]. None of these studies showed differences in plasma DHA levels after supplementing with DHA-containing phospholipids as compared to DHA-containing triglycerides, although some studies did reveal superiority of DHA-containing phospholipids on DHA levels in other tissues, such as RBC [21, 53] and the brain [11, 53]. Nevertheless, most studies concluded that the absorption into the bloodstream is independent of type of DHA (as phospholipid or as triglyceride). The present study actually shows that supplementation of lecithin, i.e., phospholipids, further increases the rise in systemic DHA induced by dietary DHA supplementation.

It should be noted that the present experiments were each conducted under different experimental conditions. Most important differences were the amounts of lecithin supplemented to the diet (0.4 *vs.* 1.0 %), supplementation periods (2 *vs.* 3 weeks), DHA-containing oils (vegetable algae DHA oil *vs.* fish oil), rat strain (Wistar *vs*. Sprague–Dawley), diet base (grain-based *vs.* AIN-93M-based), and diet manufacturer (Research Diet Services and Ssniff Spezialdiäten). However, despite the differences in experimental conditions, the results from both experiments are highly consistent with some small differences in effects sizes. For example, in Experiment B a significant additional effect of dietary lecithin supplementation on top of dietary DHA supplementation on relative plasma DHA level and quantitative plasma DHA concentration were observed, whereas in Experiment A this additional effect was only observed on relative plasma DHA level. Overall, the experiments demonstrate a substantial effect of combined dietary supplementation of lecithin and DHA on plasma and RBC levels of DHA, EPA, n-3 PUFA, and n-6 PUFA.

Important to consider when extrapolating the current results to a human setting is the physiological difference between rats and human. Rats have a continuous release of hepatic bile as opposed to burst release of gall bile in humans. Whether this physiological difference has an impact on the effects of lecithin supplementation is difficult to predict. In addition, for a better interpretation of the current results, future preclinical experiments should also incorporate measurements in other important lipid pools that either influence the plasma lipid pool (e.g., the liver) or that are influenced by the plasma lipid pool (e.g., the brain).

Dietary DHA-containing oils and crude lecithin have synergistic effects on increasing plasma and RBC n-3 PUFA levels, including DHA and EPA. By increasing the systemic availability of dietary DHA, dietary lecithin may increase the efficacy of DHA supplementation when their intake is combined. Lecithin itself also raises plasma choline concentration. Therefore, combined supplementation of lecithin and DHA may be relevant in conditions that are associated with lower levels of plasma DHA and choline.

## Abbreviations

DHA: Docosahexaenoic acid
EPA: Eicosapentaenoic acid
GC: Gas chromatography
HPLC: High performance liquid chromatography
MUFA: Monounsaturated fatty acid(s)
n-3: Omega-3
n-6: Omega-6
PUFA: Polyunsaturated fatty acid(s)
PtdCho: Phosphatidylcholine
PtdEtn: Phosphatidylethanolamine
PtdIns: Phosphatidylinositol
RBC: Red blood cell(s)
SFA: Saturated fatty acid(s)
